# B-cell maturation antigen expression across hematologic cancers: a systematic literature review

**DOI:** 10.1038/s41408-020-0337-y

**Published:** 2020-06-30

**Authors:** Ahmet Dogan, David Siegel, Nguyet Tran, Alan Fu, Jessica Fowler, Rajesh Belani, Ola Landgren

**Affiliations:** 1 Memorial Sloan Kettering Cancer Center, New York, NY USA; 2 John Theurer Cancer Center at Hackensack University Medical Center, Hackensack, NJ USA; 3 Amgen, Inc., Thousand Oaks, CA USA

**Keywords:** Cancer, Diseases, Health care, Medical research

## Abstract

B-cell maturation antigen (BCMA) plays a critical role in regulating B-cell proliferation and survival. There is evidence for BCMA expression in various hematologic malignancies, suggesting that BCMA may play an important role as a biomarker or therapeutic target in these diseases. Given advances in understanding the role of BCMA in B-cell development and the promise of BCMA as a therapeutic target, a systematic review is needed to rigorously assess the evidence for BCMA expression and identify areas of consensus and future research. The objective of this review was to summarize the evidence on BCMA protein and mRNA expression across hematologic malignancies. Using a PubMed database search up to 28 August 2019, a systematic literature review of publications reporting BCMA expression in patients with hematologic malignancies was conducted. Data from published congress abstracts presented at the American Society of Clinical Oncology and the American Society of Hematology were also searched. Studies that assessed BCMA expression (protein or mRNA) in patients of any age with hematologic malignancies were included. A total of 21 studies met inclusion criteria and were included in the review. BCMA was expressed in several hematologic malignancies, including multiple myeloma (MM), chronic lymphocytic leukemia, acute B-lymphoblastic leukemia, non-Hodgkin lymphoma (NHL), and Hodgkin lymphoma. BCMA was expressed at uniformly high levels across all 13 MM studies and at low to moderate levels in acute myeloid leukemia and acute lymphoblastic leukemia. These results suggest that BCMA is a relevant target in MM as well as in a subset of B-cell leukemia. BCMA expression in Hodgkin lymphoma and NHL varied across studies, and further research is needed to determine the utility of BCMA as an antibody target and biomarker in these diseases. Differences in sample type, timing of sample collection, and laboratory technique used may have affected the reporting of BCMA levels.

## Introduction

B-cell maturation antigen (BCMA) plays a critical role in regulating B-cell proliferation and survival, as well as differentiation into plasma cells^1,2^. BCMA is shed from the surface of plasma cells via γ-secretase–mediated cleavage, resulting in a soluble form (sBCMA)^3^. The effects of BCMA expression have been studied extensively in multiple myeloma (MM), and BCMA can be targeted to achieve anti-tumor effects in MM patients^4^. Clinical trials of anti-BCMA therapies, including antibody-drug conjugates, BiTE® (Bispecific T-cell engager) molecules, and chimeric antigen receptor T (CAR T) cell therapy, have demonstrated promising efficacy^5–9^. There is also evidence of BCMA expression in other hematologic malignancies, including B-cell leukemias and lymphomas^10,11^.

There is currently a lack of systematic reviews integrating knowledge of BCMA expression in hematologic malignancies. The primary objective of this review is to systematically assess the scientific literature and identify which hematologic cancers express BCMA. This review will summarize the evidence for BCMA expression levels in patients with hematologic malignancies.

## Methods

### Data collection and study identification

This review was fully compliant with the 2009 preferred reporting items for systematic reviews and Meta-Analysis guidelines for the reporting of systematic reviews^12^. A systematic literature review of English-language publications reporting BCMA expression in patients with hematologic malignancies was conducted using a PubMed database search up to 28 August 2019. Data from published congress abstracts presented at the American Society of Clinical Oncology and the American Society of Hematology were also searched. Studies that reported on patients of any age with hematologic malignancies and assessed BCMA expression (protein or mRNA) were included. Broad search terms, including terms for MM, leukemia, lymphoma, and BCMA expression, were used to capture potentially relevant literature. The full list of search terms is shown in Table 1. Reference lists of included publications, as well as reference lists of other systematic reviews, were also searched for relevant publications. Animal studies, studies using non-human and/or non-hematologic cell lines, studies investigating non-hematologic conditions, review articles, comments, editorials, letters, guidelines, legal cases, debates, newspaper articles, opinions, protocols, workshops, patient education brochures, and non-English publications were excluded.

**Table 1:** Publication database search strategies.

Pub Med search terms	Filters
“Multiple myeloma” OR “Acute lymphoblastic leukemia” OR “Acute myelogenous leukemia” OR “Chronic lymphocytic leukemia” OR “Chronic myelogenous leukemia” OR “Acute monocytic leukemia” OR “Hodgkin’s lymphoma” OR “Nodular sclerosing HL” OR “Mixed-cellularity subtype HL” OR “Diffuse large B-cell lymphoma” OR “Anaplastic large cell lymphoma” OR “Burkitt lymphoma” OR “Lymphoblastic lymphoma” OR “Mantle cell lymphoma” OR “Peripheral T-cell lymphoma” OR “Transformed follicular and transformed mucosa-associated lymphoid tissue lymphoma” OR “Follicular lymphoma” OR “Cutaneous T-cell lymphoma” OR “mycosis fungoides” OR “Sézary syndrome” OR “Lymphoplasmacytic lymphoma Waldenström macroglobulinemia” OR “Marginal zone B-cell lymphoma” OR “MALT lymphoma” OR “Small cell lymphocytic lymphoma” OR “chronic lymphocytic leukemia”) AND (“BCMA expression” OR “BCMA” OR “B-Cell Maturation Antigen” OR “TNFRSF17” OR “TNFRSF17 expression”	Species: Humans

### Study selection

Two independent reviewers from Amgen, Inc. first screened the title and abstracts returned from the search criteria and, in the event of ambiguous content relevance, proceeded with an in-depth review of the main text. Any disagreements between the reviewers with respect to content relevance was resolved by a third reviewer from Amgen, Inc.

### Data abstraction

Data including country or region, sample size, patient characteristics, hematologic malignancy studied, tumor burden, sample type, method of cell preparation/purification, laboratory technique, cutoff for BCMA positivity, level of BCMA expression (mRNA or protein) investigated, and percentage of patients with BCMA expression were extracted from studies included in this review. When data from congress abstracts were also in full-text publications, only unique data were extracted.

## Results

**Fig. 1:**
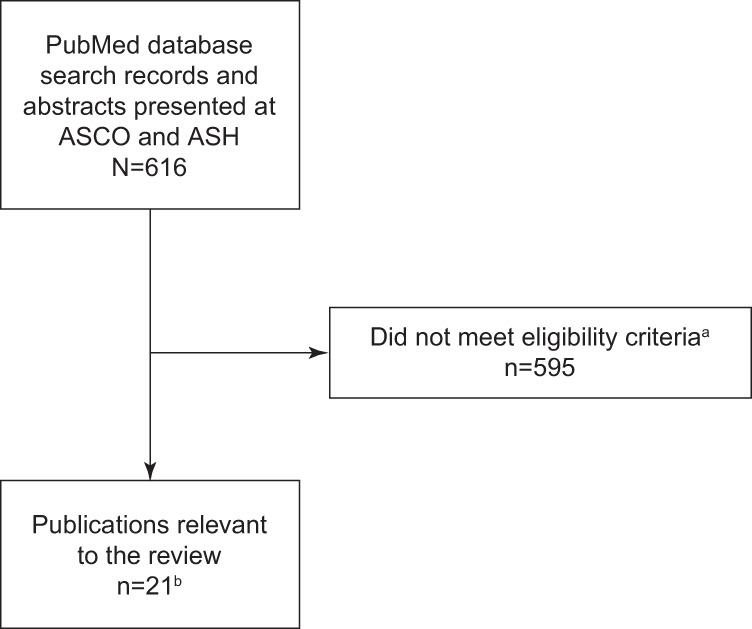
Identification of relevant publications. **a** Animal studies, studies using non-human and/or non-hematologic cell lines, studies investigating non-hematologic conditions, review articles, comments, editorials, letters, guidelines, legal cases, debates, newspaper articles, opinions, protocols, workshops, patient education brochures, and non-English publications were excluded. **b** Three studies were identified from reference lists of review publications.

Using the aforementioned criteria and search terms, the search yielded 616 studies. After excluding animal studies, 428 studies were reviewed, of which 21 met inclusion criteria; 3 studies were identified from reference lists of review publications (Fig. 1). Study information and methodology, patient characteristics, and percentage of patients with BCMA expression are summarized in Table 2 and described below. BCMA protein and mRNA expression across hematologic malignancies is summarized in Table 3, with additional details presented in the text. All studies were conducted in the United States unless otherwise specified.

**Table 2:** BCMA expression across hematologic cancers.

Study	*N*	Hematologic malignancy	Tumor burden	Sample type and method of cell preparation/purification	Laboratory technique	BCMA mRNA or protein expression (Yes/No, proportion, and expression intensity)
Seckinger et al.^13^	778 patients	Previously untreated, therapy-requiring MM, *n* = 684; relapsed MM, *n* = 94	Not reported	Patient-derived MM cells; purified using anti-CD138 microbeads	Microarray for mRNA (*n* = 712); RNA-seq for mRNA (*n* = 263); Flow cytometry for surface BCMA antigen expression (*n* = 43)	Yes (100%); median (range) surface BCMA antigen density: 1479 SABC units (42–14 055) in malignant plasma cells; 673 SABC units (189–1 713) in normal plasma cells; ≤65 SABC units (0–213) in all other normal BM cell subsets
Lee et al.^14^	70 patients	Primarily MM (including relapsed); also investigated CLL, DLBCL, FL, HL	Not reported (21 patients had ISS score and 34 patients had B2M at time of BM biopsy)	Human myeloma cell lines FFPE for IHC Incubation with FITC- or PE-conjugated anti-BCMA antibodies for flow cytometry	IHC (*n* = 64) and flow cytometry (*n* = 64) for surface BCMA antigen expression - For flow cytometry, MFI ratio (i.e., mean cell fluorescence with specific antibody compared with isotype control) was used - For IHC, the BCMA/BLIMP1 scores refers to the proportion of BLIMP + cells that were also BCMA + (scores of 0–5 correlating to 0%, 1–10%, 10–40%, 40–60%, 60–80% and >80%, respectively) Immunoassay for sBCMA concentrations (*n* = 42)	Yes (MM); 64/64 (100%) patients expressed cell surface BCMA BCMA MFI ratios ranged from 1–67 Circulating BCMA levels in serum: 3.8–1062 ng/mL (*p* < 0.0001 vs. normal controls) Varied surface BCMA expression on MM cell lines - Cell lines expressing high surface BCMA were derived from pleural fluid (H929^43^ and JIM3) and blood (MM.1s^44^]) - Cell line expressing low surface BCMA (KMM-1) was derived from plasmacytoma tissue of extramedullary origin^45^) Surface BCMA expression was low or absent in patients with smoldering/asymptomatic MM BCMA was not expressed in CLL, DLBCL, FL BCMA was expressed on plasma cells of HL but not on tumor cells
Quinn et al.^15^	34 patients	Newly diagnosed (*n* = 6) and relapsed MM (1st relapse, *n* = 10; 2nd relapse, *n* = 13; 3rd relapse, *n* = 4; 4th relapse, *n* = 1)	Not reported	Patient-derived, CD138 + purified primary MM cells	Flow cytometry for cell surface BCMA expression	Yes; 28/34 (82%) patients expressed varying BCMA levels on primary MM cells (mostly low to moderate expression; median MFIr, 1.9 [range, 0.8–13.0])
Tai et al.^16^	NA	MM	Not reported	Human MM cell lines and primary MM tumor cells	Real-time qRT-PCR for mRNA (normal donors, *n* = 3; MM patients, *n* = 5) Flow cytometry for cell surface BCMA expression	Yes; BCMA mRNA was upregulated in CD138-purified plasma cells from MM patients (*n* = 5) vs. normal donors (*n* = 3) (*p* < 0.04) BCMA membrane expression was universally detected by a novel anti-BCMA antibody in MM cell lines and CD138+ patient MM cells
Carpenter et al.^17^	6 patients	MM	Not reported	BCMA + MM cell lines; Patient-derived FFPE tissue samples and PMBCs	Real-time qPCR for mRNA IHC and flow cytometry for cell surface BCMA expression	Yes; uniform BCMA cell surface expression was detected on primary MM cells from 5 of 5 (100%) patients Cell surface BCMA expression was detected in MM cell lines H929, U266, and RPMI8226
Khattar et al.^10^	672 routine tissue and decalcified BM biopsies	B-cell lymphomas, *n* = 278; plasma cell neoplasms, *n* = 175 (MM, *n* = 162; plasmacytoma, *n* = 13); HL, *n* = 44; T-cell lymphoma, *n* = 57; myeloid and lymphoblastic lymphoma/ leukemia, *n* = 31; normal lymphoid tissues, *n* = 45	B-cell lymphomas included low grade (CLL/SLL, MCL, marginal zone lymphoma, lymphoplasmacytic lymphoma) and intermediate and high grade (DLBCL, Burkitt, plasmablastic) lymphomas	Patient-derived tissue (FFPE) and decalcified BM biopsies	IHC for cell surface BCMA expression Scoring system based on predominant pattern (Golgi, membranous, Golgi and membranous), intensity (1+, 2+, 3+) and percentage BCMA expression in neoplastic cells (0–100%); positive BCMA expression cutoff, >20%	Yes (strength variable); positive BCMA expression in: 0/57 T-cell NHL 108/278 B-cell lymphoma 1/31 myeloid and lymphoblastic leukemia, 0/44 HL, 162/162 plasma cell neoplasms 97% plasma cell neoplasms were BCMA-positive; predominant Golgi and membranous pattern, 3+ intensity DLBCL and low-grade B-cell lymphomas: predominant membranous pattern, 2+ to 3+ intensity High grade lymphomas: membranous and Golgi pattern, 3+ intensity Burkitt lymphoma (88%) and plasmablastic lymphoma (100%): membranous and Golgi pattern, 3+ intensity T-cell lymphoma, HL, myeloid and lymphoblastic lymphoma/leukemia: BCMA negative
Kinneer et al.^18^	22 patients	MM	Not reported	Primary patient BM samples Patient serum samples	Flow cytometry for cell surface BCMA expression ELISA for sBCMA	Yes; BCMA was detected on the surface of both MM cells (defined as CD19+ CD138−) and myeloma precursor cells (defined as CD19− CD138+) in all samples (*n* = 8) sBCMA concentrations (average): 269 ng/mL in patients with MM (*n* = 22), 71 ng/mL in healthy donor controls (*n* = 29)
Friedman et al.^21^	66 patients	MM (*n* = 29), HL (*n* = 7), MCL (*n* = 7), marginal zone lymphoma (*n* = 7), DLBCL (*n* = 7), FL (*n* = 7); primary B-CLL (*n* = 2)	Not reported	Patient-derived MM and lymphoma cell lines Patient-derived biopsies (FFPE) derived from BM (MM) and various tissues (lymphomas) PBMCs isolated from whole blood and cryopreserved (B-CLL)	Flow cytometry and antibody binding capacity assay for cell surface BCMA receptor density IHC for cell surface BCMA expression (BCMA staining intensity and frequency were measured on 0–4 point scales [0 = negative to 4 = intense/frequent])	Yes; MM**:** - Cell surface BCMA expressed in 29/29 (100%) patient biopsies at variable frequency - Cell surface BCMA expression moderate (score = 2) and occasional to frequent (score = 3.5) in the U226-B1 cell line; cell surface BCMA expression strong (score = 3) and frequent (score = 4) in the RPMI-8226 cell line - BCMA receptor density was not determined in the U226-B1 cell line, and was 12590 in the RPMI-8226 cell line - BCMA+ cells represented >50% tumor tissue area in 41% of biopsies NHL and HL: - Cell surface BCMA expressed in 34/35 (97%) patient biopsies (weak-to-moderate, *n* = 5; moderate, 24; moderate-to-strong, *n* = 4) - BCMA + cells were observed in >5% of tumor cells in 57% of HL and 18% of NHL samples - No lymphoma biopsy displayed >30% BCMA + cells *NHL:* - Cell surface BCMA expressed in 28/28 (100%) patient biopsies (intensity range: weak/moderate to moderate/strong [score = 1.5–2.5]) - Cell surface BCMA expressed in 4/9 (44%) cell lines (intensity range: weak to intense [score = 1.5– 3.5]; frequency range: rare to frequent [score = 2.5–4]) - BCMA+ cells observed in >5% tumor cells in 18% of biopsies ***B-CLL:*** - Cell surface BCMA expressed weakly (10% BCMA+) in 1/2 (50%) patient-derived PBMC samples ***HL:*** - Cell surface BCMA expressed in 6/7 (86%) patient biopsies (intensity range: weak/moderate to moderate/strong (score = 1.5–2.5) - Cell surface BCMA expressed in 1/2 (50%) cell lines (intensity range: weak to moderate [score = 1.5]; frequency range: rare to occasional [score = 2.5]) - BCMA+ cells observed in >5% tumor cells in 57% of biopsies
Bluhm et al.^24^	8 patients	MM, *n* = 3 patients B-NHL, *n* = 5 patients (MCL, *n* = 1; CLL, *n* = 2; FL, *n* = 1; DLBCL, *n* = 1)	Not reported	MM: MM cell lines (MM.1 S, NCI-H929, and OPM-2; frozen aliquots) Fresh or archived patient-derived CD138 + MM cells (PBMCs, *n* = 1; BM, *n* = 2) purified with Biocoll gradient B-NHL: B-NHL cell lines representative of FL (DOHH-2 and SC-1), germinal center B-cell DLBCL (SU-DHL-4 and OCI-Ly7), CLL (MEC-1), and MCL (JeKo-1) (frozen aliquots) Patient-derived PBMCs (MCL, CLL, FL) and patient-derived xenograft DLBCL sample B-ALL and T-ALL: Cell lines (frozen aliquots)	Flow cytometry for cell surface BCMA expression QuantiBRITE PE calibration beads for determining BCMA density/cell	Yes MM: 3/3 (100%) MM cell lines displayed substantial BCMA surface expression (4 300–25 000 BCMA molecules/cell), and 3/3 (100%) primary MM samples also expressed BCMA robustly B-NHL: *B-NHL cell lines:* positive BCMA expression in 6/6 (100%) B-NHL cell lines (DLBCL and FL lines: 400–500 molecules; MCL line: <100 molecules) *B-NHL primary cells:* positive BCMA expression in 4/5 (80%) samples (mantle cell lymphoma, 115 receptors/cell; B-CLL, 35–40; DLBCL, 3400); primary FL cells were BCMA-negative B-ALL and T-ALL: BCMA-negative
Sanchez et al.^25^	272 patients	MM 209 MM (including previously treated with progressive disease [*n* = 80], previously treated with responsive disease [*n* = 79], and untreated, newly diagnosed MM [*n* = 50]), 23 MGUS, 40 age-matched controls	Newly diagnosed MM (*n* = 50): ISS stage 1: *n* = 30 ISS stage 2: *n* = 12 ISS stage 3: *n* = 7 ISS stage unknown: *n* = 1	Patient-derived serum and supernatant	ELISA for sBCMA levels	Yes; median sBCMA concentrations in MM 13.87 ng/mL, MGUS 5.30 ng/mL, and healthy controls 2.57 ng/mL; *p* = 0.0157 MM vs. MGUS and *p* < 0.0001 MM vs. control, respectively Median sBCMA concentrations in indolent MM (*n* = 16; 11.60 ng/mL) lower than active MM (*n* = 34; 17.79 ng/mL)
Sanchez et al.^26^	626 patients	MM (Igκ MM, *n* = 117; Igλ MM, *n* = 62; IgG MM, *n* = 313; IgA MM, *n* = 134)	Not reported	Patient-derived serum from PB	Nephelometry for serum IgA and IgG levels Hevylite assays for levels of BCMA, uninvolved IgG, and heavy chain-light chain isoform pairs	Yes (exact BCMA levels not reported) Inverse correlation between sBCMA levels and the following: IgGλ levels in patients with IgGκ MM (*p* < 0.0001); IgGκ levels in patients with IgGλ MM (*p* = 0.0006); uninvolved IgA in patients with IgG MM (*p* < 0.0001); uninvolved IgG levels in patients with IgA MM (*p* < 0.0001)
Ghermezi et al.^27^	243 patients (3 non-secretory patients)	MM (including SMM, untreated active MM, and relapsed MM)	ISS stage: ISS stage 1: *n* = 85 (35%) ISS stage 2: *n* = 42 (17%) ISS 3 stage: *n* = 54 (22%) ISS stage not classified: 61 (25%) Mean (range) β-2 microglobulin: 3.5 mg/L (1.0–26.4)	Patient-derived serum (thawed from frozen); for PFS and OS correlational analyses, samples were collected prior to the start of a new treatment regimen (prior to initiation of first treatment, *n* = 38; prior to initiation of salvage therapy, *n* = 146)	ELISA for sBCMA levels (age-matched healthy donors, *n* = 43; SMM, *n* = 46; untreated active MM, *n* = 44)	Yes; Median sBCMA: healthy donors, 36.8 ng/mL; SMM, 88.9 ng/mL (*p* < 0.0001 vs. healthy donors); active untreated MM, 505.9 ng/mL (*p* < 0.0001 vs. healthy donors and *p* < 0.0001 vs. SMM patients) Higher-than-median sBCMA levels were predictive of shorter PFS (*p* = 0.0006) and OS (*p* = 0.0108)
Bellucci et al.^28^	196 patients	Primary MM: *n* = 33; MGUS: *n* = 5; CLL: *n* = 30; T-ALL: *n* = 33; B-ALL: *n* = 90; normal plasma cells: *n* = 5	Not reported	Patient-derived primary tumor samples (MM and B-cell lineage leukemias); purified CD138 + plasma cells (MGUS)	Microarray for BCMA gene expression	Yes (100%); BCMA gene was highly expressed in all myeloma samples BCMA expression was lower in patients with MGUS, but the difference was not statistically significant vs. normal plasma cells or myeloma cells BCMA expression was significantly lower in B-CLL and pre-B ALL vs. normal plasma cells or myeloma cells (*p* < 0.001); these low levels were similar to the BCMA level in T-ALL
Maia et al.^11^	72 patients (BCMA mRNA expression assessed in 36 patients and 6 B-ALL lines)	B-cell ALL	Not reported	Patient-derived B-ALL cells collected from BM or PB (including 21 BM, 22 PB, and 6 BM + PB samples), and B-ALL cell lines (*n* = 6)	PCR for BCMA mRNA Flow cytometry for cell surface BCMA expression	Yes; BCMA expressed in 4/6 (67%) B-ALL lines and in 36/36 (100%) primary B-ALL specimens Cell surface BCMA expression in patients (*n* = 23): mean 27%, range 1–97% Cell surface BCMA expression in B-ALL cell lines (*n* = 6): mean 2%, range 1–5%
Bolkun et al.^29^	24	AML (newly diagnosed, untreated); 12 patients achieved CR after first induction and 12 were NR	Minimally differentiated (M0): *n* = 7 Without maturation (M1): *n* = 6 With maturation (M2): *n* = 5 Acute myelomonocytic leukemia (M4): *n* = 6	Patient-derived PBMCs isolated before induction treatment using density gradient centrifugation	qPCR for mRNA Immunofluorescence staining and flow cytometry for cell surface BCMA expression	Yes; BCMA mRNA expression was higher in CR vs. NR patients (8.16 ± 5.20 vs. 0.12 ± 0.03, *p* = 0.0127) Baseline BCMA protein expression on CD33+ AML blasts was detected in CR but not NR patients
Sun et al.^30^	43 patients (20 patients with untreated ALL assessed for BCMA expression)	Pediatric B-ALL (newly diagnosed and untreated, *n* = 20; ALL in complete or partial remission, *n* = 23)	Not reported	Patient-derived BMCs	Real-time qPCR for mRNA	Yes; BCMA was expressed in 20/20 (100%) of patients with newly diagnosed, untreated B-ALL BCMA mRNA levels were significantly higher in untreated B-ALL patients vs normal controls (*p* < 0.001)
Novak et al.^31^	100	NHL, including small lymphocytic lymphoma (B-CLL/SLL), FL, DLC lymphoma, MCL, and marginal zone lymphoma	Lugano stage: Stage I–II: *n* = 38 Stage III–IV: *n* = 62	Patient-derived BMCs from lymph node biopsies	Flow cytometry for cell surface BCMA expression	No (0%); BCMA was undetectable in NHL B cells
Elsawa et al.^32^	66 patients (41 healthy, 25 WM)	WM (12 patients with WM assessed)	Not reported	Malignant B cells from a WM cell line as well as from patient-derived BM and tissue biopsy mononuclear cells	Flow cytometry; the following cutoffs were used to define BCMA expression: high expression, MFI > 21; intermediate expression, MFI 7–20; low expression, MFI 2–6; no expression, MFI < 2	Yes; BCMA was detectable in 8/12 (67%) patients at variable levels (intermediate expression [MFI, 7–20], *n* = 2; low expression [MFI, 2–6], *n* = 6)
Ferrer et al.^33^	42 patients	CLL	Binet stages: Stage A: *n* = 36 Stage B: *n* = 6 Stage C: *n* = 0 Mean (range) β-2 microglobulin; 2.2 (1.2–9.8 mg/L)^a^	Patient-derived B cells isolated from cryopreserved PBMCs	Flow cytometry for cell surface BCMA expression	Yes; BCMA MFI was significantly higher on CLL cells (553.8 [50%]) vs. normal B cells (166.2 [13%]) (*p* < 0.001)
Cols et al.^34^	21 patients	CLL	Not reported	Patient-derived B cells isolated from PBMCs	Quantitative RT-PCR for mRNA Flow cytometry for cell surface BCMA expression	Yes; BCMA was expressed on malignant B cells from 9 (43%) CLL cases
Chiu et al.^35^	15 patients	HL	Not reported	Patient-derived frozen tissue samples Cell lines (*n* = 5) derived from primary HRS cells of classical HL tumors	RT-PCR for BCMA mRNA Immunoblot for BCMA protein Flow cytometry for cell surface BCMA expression in CD30 + HRS cells Immunofluorescence staining for patient-derived frozen tissue samples	Yes; BCMA was expressed in 10/15 (67%) primary CD30 + HRS cells (+/−staining, *n* = 2) Cell surface BCMA was expressed in 4/5 (80%) cell lines (weak +/− staining, *n* = 1) 4/5 (80%) HRS cells contained BCMA transcripts and proteins

*AML* acute myeloid leukemia, *ALL* acute lymphocytic leukemia, BCMA B-cell maturation antigen, *BCR*, B-cell receptor, *BM* bone marrow, *BMC* blood mononuclear cell, *CLL* chronic lymphocytic leukemia, *CR* complete response, *DLBCL* diffuse large B-cell lymphoma, *DLC* diffuse large cell, *EU* European Union, *FFPE* formalin-fixed paraffin-embedded, *FL* follicular lymphoma, *HRS* Hodgkin and Reed-Sternberg, *Igκ* immunoglobulin κ, *Igλ* immunoglobulin λ, *IgA* immunoglobulin A, *IgG* immunoglobulin G, *ISS* International Staging System, *MCL* mantle cell lymphoma, *MFI* mean fluorescence intensity, *MFIr* median fluorescence intensity ratio, *MGUS* monoclonal gammopathy of undetermined significance, *MM* multiple myeloma, *NA* not applicable, *NHL* non-Hodgkin lymphoma, *NR* no further response to induction therapy, *PB* peripheral blood, *PBMC* peripheral blood mononuclear cells, *qPCR* quantitative polymerase chain reaction, *qRT-PCR* quantitative reverse transcription–polymerase chain reaction, *SABC* specific antibody-binding capacity, *sBCMA* serum BCMA, *SLL* small lymphocytic leukemia, *SMM* smoldering multiple myeloma, *UK* United Kingdom, *WM* Waldenstrom macroglobulinemia.

^a^Data for β-2 microglobulin is assumed to be mean (range) although this was not specified in the primary publication.

**Table 3:** Reference guide for BCMA protein and mRNA expression across hematologic malignancies.

Tumor type	Cell surface BCMA	sBCMA	BCMA mRNA
Multiple myeloma	+^10,13,14,18–23^	+^14,21,24–26^	+^13,19,20,27^
T-ALL	−^23^	NA	+^a,27^
B-ALL	−^23^	NA	+^11,27,29^
Mature B-cell neoplasms	+^10,22^ −^30^	NA	NA
B-cell lymphoma	+^10^	NA	NA
DLBCL	−^14,30^ +^10,22,23^	NA	NA
Plasmablastic lymphoma	+^10^	NA	NA
Burkitt’s lymphoma	+^10,22^	NA	
CLL or CLL/SLL	−^14,30^ +^22,23,32,33^	NA	+^a,27,33^
FL	−^14,30^ +^10,22,23^	NA	NA
MCL	−^30^ +^22,23^	NA	NA
Marginal zone lymphoma	−^30^ +^22^	NA	NA
Waldenstrom’s macroglobulinemia	+^31^	NA	NA
HL^b^	−^10,14^ +^22,34^	NA	+^34^
T-cell lymphoma	−^10^	NA	NA
Myeloid and lymphoblastic lymphoma/leukemia	−^10^	NA	NA
AML^c^	+^28^	NA	+^28^

+ positive BCMA expression, − negative BCMA expression, *AML* acute myeloid leukemia, *B-ALL* B-cell acute lymphoblastic leukemia, BCMA B-cell maturation antigen, *CLL* chronic lymphocytic leukemia, *DLBCL* diffuse large B-cell lymphoma, *HL* Hodgkin lymphoma, *FL* follicular lymphoma, *MCL* mantle cell lymphoma, *NA* not applicable, *NHL* non-Hodgkin lymphoma, *sBCMA* serum BCMA, *SLL* small lymphocytic leukemia, *T-ALL* T-cell acute lymphocytic leukemia, *WM* Waldenstrom’s macroglobulinemia.

^a^Very low expression reported in Bellucci et al.^28^.

^b^In Lee^14^, BCMA was expressed on plasma cells of HL but not on tumor cells.

cIn Bolkun et al.^29^, BCMA protein expression on CD33 + AML blasts was detected in patients who experienced complete remission after first induction, but not in non-responders.

### BCMA expression in MM

Of the 13 studies evaluating BCMA expression in MM, all reported detectable BCMA expression.

### Cell surface and intracellular BCMA protein expression

In a European study by Seckinger et al.^13^, BCMA was identified as a potential therapeutic target in newly diagnosed MM (NDMM) or relapsed MM. Cell surface BCMA expression was measured as specific antibody-binding capacity (SABC) units using multidimensional flow cytometry. Samples from 31 previously untreated MM patients and 12 patients with relapsed MM were analyzed. Surface BCMA was expressed on malignant plasma cells of previously untreated and relapsed patients with MM (median of 1479 SABC units; range, 42–14,055). The expression was higher on the malignant cells compared with both normal plasma cells (median of 673 SABC units; range, 189–173) and other bone marrow (BM) cells subsets (median ≤ 65 SABC units; range 0–213). Surface BCMA expression on plasma cells (normal or malignant) was significantly higher (*p* < 0.001) than that of the normal, non-plasma cells.

A United Kingdom (UK) study of 70 patients with MM found that BCMA expression is maintained through relapse, extramedullary spread, and residual disease following therapy^14^. BCMA expression was assessed in patient-derived BM aspirate MM cells using flow cytometry (*n* = 64) and immunohistochemistry (IHC; *n* = 64). All (64/64) patients with symptomatic MM expressed cell surface and intracellular BCMA at varying levels by IHC. The median BCMA mean fluorescence intensity (MFI) ratio was 4.1 (range, 1–67) as measured by flow cytometry. Higher levels of cell surface BCMA were associated with shorter progression-free survival (PFS) and overall survival (OS) in patients with both high- or standard-risk cytogenetics, indicating that cell surface BCMA levels may have independent prognostic value when used with conventional cytogenetic assessments. Cell surface BCMA expression was also assessed in human MM cell lines by flow cytometry, and results are summarized in Table 2.

In a UK study of patients with NDMM (*n* = 6) and relapsed (*n* = 28) MM, cell surface BCMA levels were analyzed in primary MM cells from the BM of patients who were off therapy^15^. All 28 evaluable patients expressed BCMA; levels generally ranged from low to moderate. The median fluorescence intensity ratio was 1.9 (range, 0.8–13.0), as analyzed by flow cytometry.

Tai et al.^16^ found that BCMA is universally expressed on the MM cell surface and its expression is restricted to plasma cells. Cell surface BCMA protein expression was measured by MFI using flow cytometry analysis. BCMA membrane expression was universally detected by an anti-BCMA antibody in MM cell lines and CD138+ patient-derived MM cells (*n* = 12). Carpenter et al.^17^ assessed expression in BCMA-positive MM cell lines, formalin-fixed paraffin-embedded (FFPE) tissue samples, and peripheral blood mononuclear cells (PBMCs) derived from six patients with MM. Cell surface BCMA was measured as the number of events using flow cytometry, and IHC staining was performed with BCMA and isotype-matched control antibodies on consecutive sections. Using flow cytometry analysis, BCMA expression was detected on MM cell lines but not on other types of cell lines, including primary CD34+ hematopoietic cells. BCMA was uniformly expressed on the surface of primary MM cells all patients (*n* = 5), as determined by flow cytometry or IHC.

Khattar et al.^10^ evaluated cell surface and intracellular BCMA expression in 672 biopsies from normal lymphoid tissues and from patients with various hematologic malignancies, including MM, using IHC staining. FFPE tissue and decalcified BM biopsies were collected from patients with plasma cell neoplasms, including normal lymphoid tissues (*n* = 45), MM (*n* = 162), and plasmacytoma (*n* = 13). B-cell lineage malignancies and plasma cell neoplasms had high BCMA expression levels. All MM samples and 97% of plasma cell neoplasms were BCMA-positive and had a membranous and Golgi staining pattern with strong (3+) intensity.

Kinneer et al.^18^ analyzed BCMA expression on the surface of MM cells and myeloma progenitor cells (MPCs) in primary BM samples (*n* = 8) of MM patients. Unlike mature MM cells, MPCs lack a mature plasma cell phenotype and are not fully differentiated^19^. However, MPCs can transfer the disease and predict a poorer patient response to stem cell transplant and proteasome inhibitor treatment^20^, making them an important cell population to characterize. Using flow cytometry, histograms were generated on the MPCs and the MM plasma cell population. BCMA was detected on the cell surface of both MM cells and MPCs of all samples analyzed.

Friedman et al.^21^ evaluated cell surface BCMA expression in 2 MM cell lines derived from peripheral blood (PB) (U266-B1^22^, RPMI-8226^23^) and in 29 MM BM biopsies. BCMA expression in cell lines is summarized in Table 2. For BM samples from patients with MM, IHC demonstrated that 29/29 (100%) samples expressed BCMA, although the percentage of sample staining positive was variable. BCMA-positive cells represented over half of the tumor tissue area in 41% of the biopsies.

In a German study by Bluhm et al.^24^, cell surface BCMA expression was assessed in 3 MM cell lines and in primary, patient-derived PBMCs (*n* = 1 patient) and BM-derived cells (*n* = 2 patients) using flow cytometry. The MM cell lines displayed substantial BCMA surface expression (mean: 4300–25,000 BCMA molecules/cell), and primary MM cells also expressed BCMA robustly.

### sBCMA levels

In a study of 209 patients with MM, sBCMA levels in NDMM patients (*n* = 50; median 13.87 ng/mL) were significantly higher than in age- and gender-matched healthy controls (*n* = 40; median 2.57 ng/mL; *p* < 0.0001) and in patients with monoclonal gammopathy of undetermined significance (MGUS; *n* = 23; median 5.30 ng/mL; *p* = 0.0157)^25^. Patients with indolent MM (*n* = 16) had lower sBCMA levels (median 11.60 ng/mL) than those with active MM (*n* = 34; median 17.79 ng/mL). In addition, BCMA levels in patients with MM correlated with clinical response and OS. Responsive (≥partial response [PR]) patients (*n* = 80 samples) had lower sBCMA levels than patients (*n* = 79 samples) with progressive disease (median 4.06 ng/mL vs. 19.76 ng/mL, respectively; *p* = 0.0038). OS was significantly shorter in MM patients (*n* = 162) who had BCMA levels above the median concentration (10.85 ng/mL) vs. those with levels below the median (*p* = 0.0014). A subsequent study by Sanchez et al.^26^ showed that sBCMA levels inversely correlated with uninvolved polyclonal antibody production in patients with MM, suggesting a possible sBCMA-mediated mechanism of immune deficiency in these patients. In addition, BCMA-BAFF complexes were detected in PB-derived sera of patients with MM (*n* = 12 total), as determined by ELISA.

Further building upon the Sanchez et al. studies^25,26^, Ghermezi et al.^27^ identified sBCMA as a biomarker that can monitor and predict outcomes for MM patients. Serum was collected from 243 patients with MM and sBCMA levels were determined by ELISA. sBCMA levels were higher among patients with smoldering MM (SMM; *n* = 46; 88.9 ng/mL [*p* < 0.0001 vs. healthy donors]) and untreated active MM (*n* = 44; 505.9 ng/mL [*p* < 0.0001 vs. healthy donors and *p* < 0.0001 vs. SMM patients]) than in age-matched healthy donors (*n* = 43; median sBCMA of 36.8 ng/mL). In addition, sBCMA levels correlated with the proportion of plasma cells in BM biopsies (Spearman’s rho = 0.710; *p* < 0.001), clinical status (complete response [CR] vs. PR, *p* = 0.0374; CR vs. progressive disease, *p* < 0.0001), and M-protein levels. Furthermore, higher-than-median sBCMA levels were predictive of a shorter PFS (*p* = 0.0006) and OS (*p* = 0.0108). sBCMA levels also correlated with changes in M-protein and serum-free light chain levels in individual patients with MM.

In the study by Lee et al.^14^, sBCMA in patient samples (*n* = 42) ranged from 3.8–1 062 ng/mL (*p* < 0.0001 vs. normal controls), with no difference between NDMM patients vs. those with relapsed disease. In contrast to the reports by Sanchez et al.^25^ and Ghermezi et al.^25^, there was no association between sBCMA level and treatment response or survival.

The study by Kinneer et al.^18^ measured sBCMA concentrations in serum samples from patients with MM and age- and sex-matched healthy donors. As determined by ELISA, sBCMA levels were higher in patients with MM (mean sBCMA: 269 ng/mL, *n* = 22) compared with healthy donor controls (average 71 ng/mL, *n* = 27).

### BCMA mRNA expression

In the study by Seckinger et al.^13^, BCMA mRNA expression was assessed using gene expression profiling by DNA microarray (*n* = 712) and RNA sequencing (*n* = 263) in patients with NDMM or relapsed/refractory MM. BCMA mRNA was expressed in all patient samples, supporting the observed increase in cell surface BCMA expression by flow cytometry. The study by Tai et al.^16^ used real-time quantitative reverse-transcription–polymerase chain reaction (RT-qPCR) to quantify BCMA mRNA. BCMA mRNA was upregulated in CD138-purified plasma cells from patients with MM (*n* = 5) vs. normal donors (*n* = 3) (*p* < 0.04). These findings supported the enhanced cell surface BCMA protein expression observed by flow cytometry. Plasmacytoid dendritic cells had detectable BCMA mRNA at significantly lower levels than CD138+ plasma cells (*p* < 0.005 for each paired sample) from either patients with MM or normal donors. BCMA mRNA was increased in plasmacytoid dendritic cells from patients with MM vs. normal donors (*p* < 0.03).

In the study by Carpenter et al.^17^, BCMA had a restricted expression pattern, as assessed by qPCR. The BCMA expression of a plasmacytoma sample from a patient with advanced MM (~3500 BCMA cDNA copies/10^5^ cDNA actin copies) was dramatically higher than the BCMA expression of cDNA samples from several types of normal tissues (all < 500 BCMA cDNA copies/10^5^ cDNA actin copies). These results were in line with previously described findings of increased cell surface BCMA protein, as assessed by IHC and flow cytometry.

Bellucci et al.^28^ characterized BCMA expression in primary tumor samples from MM patients (*n* = 33) and in purified CD138+ plasma cells from MGUS patients (*n* = 5) and normal plasma cells (*n* = 5) using high-density oligonucleotide microarrays. BCMA was highly expressed in all MM samples. BCMA expression was lower in patients with MGUS, but the difference was not statistically significant compared with normal plasma cells or MM cells.

#### BCMA expression in acute leukemia

Of the five studies evaluating BCMA expression in leukemia, 4/5 (80%) reported BCMA expression. BCMA expression was assessed in two studies of acute B-lymphoblastic leukemia (B-ALL), two studies that investigated both T-ALL and B-ALL, and one acute myeloid leukemia (AML) study.

### Cell surface BCMA protein expression

In a study by Maia et al.^11^ (*n* = 72), cell surface BCMA was assessed in primary B-ALL cells and B-ALL cell lines. B-ALL cells were collected from patients’ BM or PB, and included 21 BM, 22 PB, and 6 BM plus PB samples. Flow cytometry was used to assess BCMA cell surface expression in primary B-ALL (*n* = 23) and B-ALL lines (*n* = 6). Mean surface BCMA expression (percentage of cells) was 27% in patients and 2% in B-ALL cell lines.

In the study by Bluhm et al.^24^, BCMA cell surface expression was examined in 2 B-ALL cell lines (NALM6 and REH). Both cell lines were BCMA-negative, as assessed by flow cytometry.

In a Polish study of 24 patients with newly diagnosed AML, cell surface BCMA protein expression was assessed in patient-derived PBMCs^29^. Patient AML ranged from minimally differentiated to acute myelomonocytic leukemia. Twelve patients achieved complete remission (CR) after first induction and 12 were non-responders (NR). Immunofluorescence staining showed that baseline BCMA protein expression on CD33 + AML blasts was present in patients who achieved CR (16%±9% of cells) but not in patients classified as NR (<1% of cells).

In the study by Bluhm et al.^24^, BCMA was not expressed in the T-ALL cell line Jurkat, as assessed by flow cytometry.

### BCMA mRNA expression

In a Chinese study by Sun et al.^30^, plasma samples were collected from 43 pediatric patients with B-ALL. Among the enrolled patients, 20 had newly diagnosed B-ALL and 23 patients were in complete or partial remission after previous treatment. Using real-time qPCR, BCMA mRNA expression was assessed in BMCs from 20 newly diagnosed B-ALL patients and 15 normal controls. BCMA was expressed in 20 (100%) patients with newly diagnosed B-ALL and was found to be significantly higher in BMCs from patients with B-ALL compared with controls (*p* < 0.001).

In the study by Maia et al.^11^, BCMA mRNA expression was assessed in primary B-ALL cells (BM or PB; *n* = 36) and B-ALL lines (*n* = 6), using RT-PCR. BCMA was detected in 36/36 (100%) primary B-ALL specimens and in 4/6 (67%) B-ALL lines. In the study by Bolkun et al.^29^, BCMA mRNA expression was assessed in patient-derived PBMCs from 24 patients with newly diagnosed AML^29^. BCMA mRNA expression was higher in CR vs. NR patients (8.16 ± 5.20 vs. 0.12 ± 0.03, *p* = 0.0127), as determined by qPCR.

In the study by Bellucci et al.^28^, BCMA expression was characterized in primary tumor samples from patients with T-ALL (*n* = 33) and B-ALL (*n* = 90) using high-density oligonucleotide microarrays^28^. BCMA expression was significantly lower in B-ALL compared with normal plasma cells or MM cells (*p* < 0.001); these low levels were similar to the BCMA level in T-ALL.

#### BCMA expression in mature B-cell neoplasms

Of the 10 studies evaluating BCMA expression in lymphoma, six studies assessed patients with non-Hodgkin lymphoma (NHL)^24,28,31–34^, one study assessed patients with Hodgkin lymphoma (HL)^35^, and three studies assessed a mixed NHL and HL population^10,14,21^. Among these studies, 7/9 (78%) reported BCMA expression in NHL and 2/4 (50%) reported BCMA expression in HL.

### Cell surface BCMA protein expression

In the study by Khattar et al.^10^, FFPE tissue and decalcified BM biopsies were collected from patients with B-cell lymphomas (*n* = 278). T-cell lymphoma (*n* = 57), myeloid and lymphoblastic lymphoma/leukemia (*n* = 31), HL (*n* = 44) and normal lymphoid tissue (*n* = 45) samples were also collected. Diffuse large B-cell lymphoma (DLBCL) and low-grade B-cell lymphoma samples predominantly showed a membranous pattern with 2+ to 3+ staining intensity using IHC. In DLBCL, 39% of patients with the active B-cell subtype and 35% of patients with the germinal center B-cell subtype had >20% BCMA expression in neoplastic cells. There were no significant differences in the intensity and pattern of staining. Eight cases (88%) of Burkitt lymphoma and seven cases (100%) of plasmablastic lymphoma showed membranous and Golgi BCMA staining patterns with 3+ intensity. BCMA was expressed in only 1/31 (3%) samples of myeloid and lymphoblastic lymphoma/leukemia and was not expressed in any samples of T-cell lymphoma or HL.

In a study of 100 patients with NHL, cell surface BCMA expression was analyzed in BMCs from lymph node biopsies using flow cytometry^31^. NHL samples were diagnosed with either small lymphocytic lymphoma (B-CLL/SLL), follicular lymphoma (FL), DLBCL, MCL, or marginal zone lymphoma. In contrast to the study by Khattar et al.^10^, which identified intermediate to high BCMA expression in several types of NHL, BCMA was undetectable in NHL B cells.

Elsawa et al.^32^ analyzed cell surface BCMA expression in malignant B cells from a Waldenstrom’s macroglobulinemia cell line and from patient-derived BM and tissue biopsy mononuclear cells. BCMA was detectable at variable levels via flow cytometry in 8/12 (67%) patients, with 2/12 (17%) patients having intermediate expression and 6/12 (50%) patients having low expression.

Ferrer et al.^33^ investigated cell surface BCMA protein expression in 42 patients with CLL who had available cryopreserved PBMC samples at the time of diagnosis. By flow cytometry, BCMA MFI was significantly higher on CLL cells (553.8, 50% of positive cells for BCMA on CD19+ cells) than on normal B cells (166.2, 13% of positive cells for BCMA on CD19+ cells) (*p* < 0.001). BCMA expression correlated with unmutated *IGHV* genes and poor-risk cytogenetics but was not correlated with disease stage, blood lymphocyte count, ZAP70, or CD38 expression. In addition, patients with a higher BCMA expression on CLL cells had a shorter PFS compared with patients with lower BCMA expression (median, 57 months vs. 206 months; *p* = 0.021). In a study of 21 patients with CLL, cell surface BCMA was expressed on malignant B cells from 9/21 (43%) CLL cases, as assessed by flow cytometry^34^.

In the study by Friedman et al.^21^, cell surface BCMA expression was evaluated in 9 NHL cell lines (myelogenous leukemia, *n* = 2; ALL, *n* = 2; MCL, *n* = 2; Burkitt’s lymphoma, *n* = 2; B-CLL, *n* = 1) using IHC. BCMA was expressed in 4/9 (44%) cell lines. Of these, BCMA staining ranged from weak to intense (score = 1.5–3.5) and rare to frequent (score = 2.5–4). Cell surface BCMA expression was also assessed in 28 biopsies from patients with NHL (*n* = 7 each for DLBCL, MCL, FL, and mantle zone lymphoma) using IHC. BCMA was expressed in all NHL biopsies, with staining intensity ranging from weak/moderate to moderate/strong (score = 1.5–2.5). In addition, BCMA-positive cells were observed in >5% of the tumor cells in 18% of NHL patient biopsies. Cell surface BCMA expression was also evaluated in 2 HL cell lines, of which 1/2 (50%) expressed BCMA at weak to moderate intensity (score = 1.5) and rare to occasional frequency (score = 2.5). BCMA was expressed in 6/7 (86%) HL biopsies, with staining intensity ranging from weak/moderate to moderate/strong (score = 1.5–2.5). In addition, BCMA+ cells were observed in >5% of the tumor cells in 57% of HL biopsies. Furthermore, 1/2 (50%) patient-derived PBMC samples was shown to be weakly (10%) BCMA+ by flow cytometry.

The study by Bluhm et al.^24^ measured cell surface BCMA expression in B-NHL cell lines representative of FL, germinal center B-cell DLBCL, CLL, and MCL. BCMA was expressed in all 6 B-NHL cell lines. The BCMA frequencies in the DLBCL and FL cell lines ranged from 400–500 molecules, and the MCL cell line had a BCMA frequency of <100 molecules. Primary B-NHL cells (CD19 + CD20 + CD138-) from patients with MCL (*n* = 1), CLL (*n* = 2), and FL (*n* = 1), as well as a patient-derived xenograft DLBCL sample, were also assessed for BCMA expression. Patient samples were derived from PBMCs, and residual CD138+ cells were excluded from analysis. BCMA was expressed on the following primary B-NHL samples: MCL, 115 receptors/cell; B-CLL, 35–40 receptors/cell; DLBCL, 3 400 receptors/cell. Primary FL cells were BCMA-negative.

Chiu et al.^35^ evaluated cell surface BCMA expression in patients with HL. Cell lines (*n* = 5) were derived from primary Hodgkin and Reed-Sternberg (HRS) cells of classical HL tumors. Cell surface BCMA was expressed at variable levels in 4/5 (80%) cell lines, as determined by flow cytometry. BCMA protein was also expressed in 4/5 (80%) cell lines, as assessed by immunoblot. Frozen tissue samples were collected from 15 patients with classical HL, including subtypes of nodular sclerosis (11), mixed cellularity (*n* = 3), and lymphocyte depletion (*n* = 1). Primary CD30 + HRS cells from classical HL tumors expressed BCMA in 10/15 (67%) cases examined.

### BCMA mRNA expression

In the study by Cols et al.^34^, BCMA mRNA expression was measured using RT-qPCR in 21 patients with CLL*.* BCMA transcripts increased after CLL cells were incubated with CD40L, a molecule aberrantly expressed by CLL cells.

In the study by Bellucci et al.^28^, BCMA mRNA expression was characterized in primary tumor samples from patients with (CLL) (*n* = 30). As measured by high-density oligonucleotide microarrays, BCMA expression was significantly lower in B-CLL compared with normal plasma cells or MM cells (*p* < 0.001).

In the study by Chiu et al.^35^, BCMA transcript and protein were expressed in 4/5 (80%) HL cell lines, as assessed by RT-PCR^35^. In contrast to the BCMA expression results reported in Chiu et al.^35^, BCMA expression was absent in CLL, DLBCL, and FL, and on classical HL tumor cells, in the study by Lee et al.^14^.

## Discussion

**Fig. 2:**
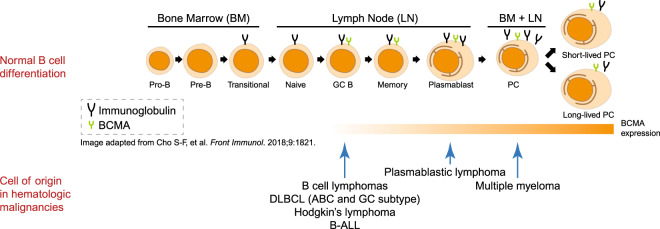
BCMA expression in normal B-cell development and in hematologic malignancies. In the top part of the figure, normal B-cell development is shown from left to right starting from pro-B-cell to the terminally differentiated plasma cell. The presence of immunoglobulin and BCMA expression is indicated in each developmental stage. In the bottom half of the figure, the cell of origin of various mature B-cell malignancies and relative intensity of BCMA expression are shown.

In this systematic review, BCMA expression was found across multiple hematologic malignancies of precursor B-cells, plasma cells or late-stage B cells (Fig. 2). In MM, all identified studies reported detectable BCMA expression in all patient samples via protein or mRNA, often at high levels. These results were consistent despite the variability in the number of included patients, laboratory techniques used to assess BCMA levels, and sample types in each study.

### MM

In the MM studies, cell surface BCMA expression was seen in MGUS, NDMM, or relapsed MM at varying levels. BCMA expression was generally lower in MGUS compared with MM^25,28^. Three studies reported higher sBCMA levels in patients with NDMM compared with controls^14,25,27^, with one of these studies finding that sBCMA levels were similar between NDMM and relapsed MM^14^.

Based on these studies, the treatment status of MM generally does not appear to have a significant influence on BCMA protein or BCMA mRNA expression. BCMA protein and mRNA expression was reported in both NDMM and relapsed MM with expression maintained in residual disease and at relapse. Patients with myeloma precursor disease (e.g., MGUS) did not have statistically significant differences in BCMA cellular expression compared with patients with MM^28^, but an increase in sBCMA correlated with more active/advanced disease stage. Most studies either combined NDMM and relapsed MM patient data or did not evaluate BCMA expression by MM treatment status; therefore, further work is needed to assess BCMA protein (cell surface and sBCMA) and mRNA expression specifically in NDMM or relapsed MM.

Several studies demonstrated that sBCMA is elevated in MM and can be used as a biomarker to predict disease response and survival^25,27^. Furthermore, sBCMA can serve as an independent prognostic marker of clinical outcomes^27^. In our analysis, four studies reported higher sBCMA levels in patients with MM compared with healthy controls^14,18,25,27^. Of these, two studies found an association between elevated sBCMA and poor clinical outcomes^25,27^. Ghermezi et al.^27^ found that sBCMA levels were markedly lower in patients with CR than in those with <CR, and both PFS and OS were shorter among patients whose sBCMA levels were above the median. Similarly, in a study by Sanchez et al, responsive (≥PR) patients had lower sBCMA levels than patients showing progressive disease and OS was shorter among patients with sBCMA levels above the median^25^. In addition, a study by Sanchez et al demonstrated an inverse correlation between sBCMA levels and levels of uninvolved polyclonal antibodies, suggesting that higher sBCMA levels can be associated with immunoparesis^26^. This evidence supports the role of BCMA as a biomarker and immuno-oncology therapy target in MM.

### Acute leukemia

For leukemia, 4/5 (80%) identified studies reported BCMA expression at varying levels^11,24,28–30^. B-ALL samples were generally BCMA-positive, with 100% of patient-derived BMCs and B-ALL cells expressing BCMA mRNA in two studies^11,30^. Based on these findings, BCMA may be a promising biomarker of response and immuno-oncology therapy target in B-ALL, but further research is warranted to determine whether anti-BCMA therapies can be effective in these malignancies. Analyses of databases (e.g., The Cancer Genome Atlas) may be particularly useful to generate additional data related to BCMA expression.

### Mature B-cell neoplasms

BCMA expression in HL and NHL varied widely across studies. In some NHL subtypes, BMCA expression or lack thereof was reported with no conflicting results, whereas BCMA expression data were conflicting in HL studies and in some subtypes of NHL. Despite these variations in BCMA expression in some NHL subtypes across studies, BCMA may still be a suitable target for mature B-NHLs. One study validated BCMA expression in DLBCL, FL, MCL, and CLL, and demonstrated that BCMA CAR T cells eradicated B-NHL cells in vitro and in vivo through target cell lysis^24^. Only 1 NHL study reported data on patient tumor burden^31^, and there were no NHL or HL studies reporting on patient treatment status. Therefore, future work is needed to determine whether BCMA expression is affected by these factors.

### BCMA expression methodology and reporting

The methodologies used to measure BCMA mRNA or cell surface protein expression may have affected the expression levels reported in each analysis. These differences in methodology could explain the conflicting results reported across some studies, particularly for HL and DLBCL (Table 2).

The majority of studies used flow cytometry and/or IHC to assess cell surface BCMA protein levels^14,17,21,29,35^. Flow cytometry has been shown to be more sensitive than IHC, particularly for specimens with few plasma cells, and can objectively quantify BCMA expression by myeloma cells^36^. An advantage of IHC is that it does not require fresh tissue. In our analysis, for HL and DLBCL, BCMA expression data were conflicting despite the use of IHC staining on patient-derived FFPE tissue samples across several studies^10,14,21^. Variation in ischemic time before fixation, duration of fixation and fixative formula, decalcification method, and sample variability may have affected IHC results^37,38^.

BCMA mRNA levels were analyzed in 8/17 (47%) of studies using several techniques, predominantly qPCR. Previous studies have shown that the cellular concentrations of proteins correlate with the quantity of their corresponding mRNAs, but the correlation is not strong^39,40^. In one report, the squared Pearson’s correlation coefficient for protein and mRNA concentrations across organisms was ~0.4, suggesting that only ~40% of the variation in protein expression can be explained by changes at the transcript level^40^. Qualitatively, in our analysis, BCMA protein and mRNA data were in agreement within studies that assessed both forms of BCMA. In 4 MM studies that measured sBCMA levels by immunoassay, sBCMA was detected in all MM patients at higher levels than in healthy controls^14,18,25,27^.

The assessment of factors that constitute BCMA positivity or negativity also varied across studies. Elsawa et al defined cutoffs of MFI > 21, 7–20, 2–6, and <2 for high, intermediate, low, and no expression, respectively, using flow cytometry in a WM cell line and patient-derived tissue^32^. Khattar et al.^10^ defined a positive BCMA cutoff of >20% using IHC in B cell lymphoma patient-derived tissue and BM biopsies. The remaining studies did not explicitly define BCMA expression cutoffs. Thus, reporting of BCMA expression may vary due to different criteria used to determine BCMA positivity or negativity in each study.

### Sample type and timing of collection

In addition to methodological differences, the sample types used in each analysis may have affected reporting of BCMA expression. The studies included in this review assessed BCMA in cell lines derived from various sources and/or patient-derived samples. As no studies in this analysis performed a head-to-head comparison of patient sample types, it is difficult to determine any potential effects of sample type on BCMA expression. The timepoint at which samples were collected may have also impacted assessment of BCMA expression, although any potential effects are challenging to evaluate as only a few studies reported these data^27,29,33^.

To better understand the utility of BCMA as a biomarker or treatment target, it will be important for future studies to longitudinally evaluate BCMA expression dynamics before and after treatment. For example, the cleaving of cell surface BCMA by γ-secretase results in shedding of the extracellular domain, leading to circulation of sBCMA^3^. Sufficient sBCMA accumulation in the BM of MM patients may inhibit BCMA CAR-T cell recognition of tumor cells^41^. Recently, γ-secretase inhibitors have been found to increase surface BCMA levels in MM cell lines and patient tumor samples, and improve tumor recognition by CAR-T cells in vitro^41^. Furthermore, in a phase 1 study of relapsed/refractory MM patients (*n* = 6 assessable patients), treatment with the γ-secretase inhibitor JSMD194 in combination with anti-BCMA CAR T cell therapy led to an ORR of 100% (5 VGPR, 1 PR)^42^.

This study had several limitations. Publication bias may have occurred if relevant ongoing or completed studies have not yet been published. Manual literature searches for BCMA expression studies may have resulted in studies being identified in a non-systematic manner. Most studies were conducted in the US or in Europe, limiting the generalizability of results to other populations. Some studies only reported mRNA data, with no protein correlative data. Based on the method with which BCMA is assessed, it is not clear whether cell surface expression is reflected, although IHC and mRNA appear to be good surrogate markers.

In conclusion, BCMA was expressed in several hematologic malignancies, including MM, CLL, ALL, NHL, and HL. BCMA appears to be a relevant target in MM, given its uniformly positive expression across studies. BCMA-targeted therapy may also be promising in patients with certain types of leukemia or lymphoma, as BCMA was also expressed in these malignancies. Further research is needed to determine the utility of BCMA as a biomarker and antibody target in hematologic malignancies for which evidence was conflicting or only 1 or a few studies were conducted^6^.

## Supplementary information

Reproducibility Checklist
